# Empirical evidence for structural balance theory in functional brain networks

**DOI:** 10.3389/fnetp.2025.1681597

**Published:** 2026-01-16

**Authors:** Majid Saberi, Abolfazl HaqiqiFar, AmirHussein Abdolalizadeh, Bratislav Misic, Ali Khatibi

**Affiliations:** 1 Headache and Orofacial Pain Effort (H.O.P.E.) Laboratory, Department of Biologic and Materials Sciences & Prosthodontics, University of Michigan School of Dentistry, Ann Arbor, MI, United States; 2 Neurosciences & Mental Health Program, The Hospital for Sick Children Research Institute, Toronto, ON, Canada; 3 Department of Physics, Faculty of Science, Bu-Ali Sina University, Hamedan, Iran; 4 Biological Psychology, Department of Psychology, School of Medicine and Health Sciences, Carl Von Ossietzky Universität Oldenburg, Oldenburg, Germany; 5 Department of Neurology and Neurosurgery, McGill University, Montreal, QC, Canada; 6 McConnell Brain Imaging Centre, Montreal Neurological Institute and Hospital, Montreal, QC, Canada; 7 Centre of Precision Rehabilitation for Spinal Pain (CPR Spine), School of Sport, Exercise and Rehabilitation Sciences, University of Birmingham, Birmingham, United Kingdom; 8 Department of Psychology, University of Bath, Bath, United Kingdom

**Keywords:** structural balance theory, higher-order interactions, dynamic functional connectivity, resting-state fMRI, brain network dynamics, brain network modeling

## Abstract

Structural balance theory, widely used in social network research, has recently been applied to brain network studies to explore how higher-order interactions relate to neural function and dysfunction. The theory is founded on the core assumption that balanced triads, representing internally consistent relationships, are intrinsically stable, while imbalanced triads, which introduce structural tension, are unstable and tend to reconfigure toward balance. Despite its promising application, these foundational assumptions have not been empirically validated in the brain. Here, we address this gap using resting-state fMRI data from the Human Connectome Project to analyze the temporal dynamics of triadic configurations. We defined two metrics: triad lifetime, the duration a triad persists, and absolute peak energy, the maximum triadic interaction strength during that time. Balanced triads showed significantly longer lifetimes and higher peak energy than imbalanced ones, consistent with their theorized stability. Imbalanced triads were more transient and weaker, reflecting structural conflict. Comparison with surrogate null models confirmed that these patterns were not random, but reflected meaningful higher-order neural organization. The joint distribution of lifetime and energy revealed two clusters of triads aligning with strong, not weak, structural balance theory. Additionally, specific transition patterns between triadic configurations, combined with lifetime profiles, shaped the non-uniform prevalence of triadic states in brain networks. Our findings provide empirical validation of structural balance theory in brain networks and introduce dynamic measures for characterizing triadic brain interactions, together offering a framework for studying the dynamics of higher-order interactions and the stability of brain networks in health and disease.

## Introduction

Understanding complex systems requires moving beyond simple pairwise interactions to capture dependencies among groups of three or more units (Battiston et al., 2021). While traditional network models rely on pairwise connections to describe system behavior, many real-world systems exhibit higher-order interactions, where multiple entities interact simultaneously (Battiston et al., 2020; Boccaletti et al., 2023). These interactions reveal emergent properties, uncover non-linear relationships, and expose topological structures that remain hidden in pairwise models (Battiston et al., 2021; Battiston et al., 2020; Boccaletti et al., 2023). By incorporating higher-order interactions, researchers can better characterize system dynamics (Boccaletti et al., 2023; Majhi et al., 2022; Grilli et al., 2017; Gao et al., 2023), phase transitions (Schawe and Hernández, 2022; Ghosh et al., 2023; Gallardo-Navarro et al., 2024; Della Rossa et al., 2023) and collective behaviors (Feng et al., 2024; Krishnagopal and Bianconi, 2023). Evidence from diverse fields underscores their significance: in ecology, many-body interactions shape species coexistence and ecosystem stability (Mayfield and Stouffer, 2017; Korkmazhan and Dunn, 2022; Bairey et al., 2016); in social systems, higher-order coordination influences group decision-making and opinion dynamics (Iacopini et al., 2019; Chowdhary et al., 2021; St-Onge et al., 2021).

In neuroscience, higher-order interactions provide a more comprehensive framework for modeling complexity in brain networks. Unlike traditional approaches that analyze pairwise neural dependencies, these methods account for the simultaneous coordination of multiple brain regions, offering deeper insights into cognitive processes (Saberi et al., 2024), individual variability (Santoro et al., 2024), and pathological conditions (Xi et al., 2024; Yang et al., 2023; Xi et al., 2023; Herzog et al., 2022; Gatica et al., 2021), using mathematical frameworks such as simplicial complexes (Saberi et al., 2024; Santoro et al., 2024; Saberi et al., 2022; Sporns and Kötter, 2004, Ji et al., 2022) and multivariate information-theoretic measures (Herzog et al., 2022; Gatica et al., 2021; Bispo et al., 2024).

Among the various mathematical frameworks used to study higher-order interactions in brain system, structural balance theory provides a unique approach for analyzing triadic relationships in functional brain networks with positive and negative connections (Saberi et al., 2024; Saberi et al., 2022). Originally introduced in social psychology by Fritz Heider (Heider, 1946; Heider, 2013), structural balance theory describes how relationships between three entities evolve toward stable or unstable configurations based on cooperative (positive) or antagonistic (negative) interactions (Cartwright and Harary, 1956). The theory has since been mathematically formalized in network science (Khanafiah and Situngkir, 2004; Estrada, 2019; Derr et al., 2018; Marvel et al., 2009), providing insights for understanding dynamics, alliance, polarization and fragmentation (Talaga et al., 2023; Minh Pham et al., 2020; Marvel et al., 2011; Antal et al., 2005) and applied across multiple domains, including social opinions and relationships (Facchetti et al., 2011; Gallo et al., 2024; Rawlings and Friedkin, 2017; Aref et al., 2020; Shebaro and Tešić, 2025), political alliances (Talaga et al., 2023; Shebaro and Tešić, 2025; Aref and Neal, 2020), ecological stability (Wey et al., 2019; Saiz et al., 2016), and brain networks (Saberi et al., 2024; Saberi et al., 2021a).

**FIGURE 1:**
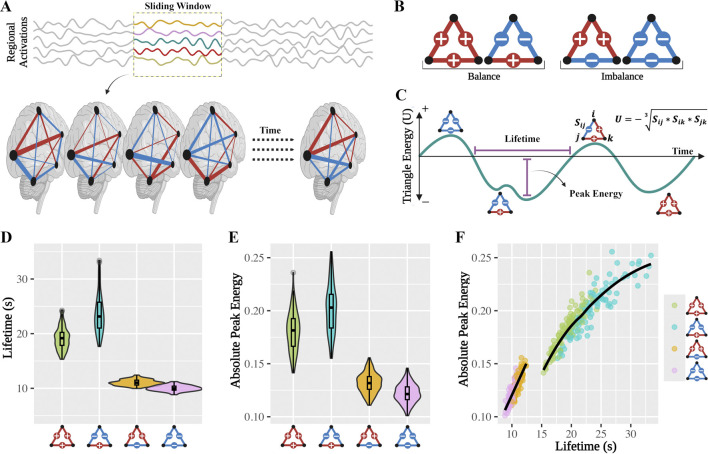
Dynamics of structural balance in brain network. **(A)** Dynamic functional connectivity was derived from regional brain activations segmented using a sliding window approach. Positive (blue) and negative (red) connections represent correlations and anti-correlations between regional time series, respectively. The width of each connection indicates the strength of the association. **(B)** Four possible signed triadic configurations were identified based on the sign of connections and classified as balanced (+++, −+−) or imbalanced (+−+, −−−) according to structural balance theory. **(C)** Schematic illustration of a triangle’s evolving state and energy over time. Two key dynamic measures are depicted: lifetime, the duration a triangle maintains a specific configuration, and peak energy, the maximum absolute energy during that period. **(D)** Violin plots showing the group-level distribution of triad lifetime for each configuration. Balanced triads exhibit significantly longer lifetimes than imbalanced ones. **(E)** Violin plots of absolute peak energy show that balanced triads have significantly higher peak energy values than imbalanced ones. **(F)** Joint distribution of lifetime and absolute peak energy across all configurations reveals two distinct clusters corresponding to balanced (green and blue) and imbalanced (orange and pink) triads.

According to the (strong) formulation of structural balance theory, triadic relationships are classified as either balanced or imbalanced (Heider, 2013; Cartwright and Harary, 1956) (Figure 1B). In a balanced triad, where the product of connection signs is positive (+++ and −+−), the triad is stable with minimized tension among its interacting elements. In contrast, imbalanced triads, where the product of connection signs is negative (−−− and +−+), introduce structural tension and instability, driving the triad toward reorganization to restore balance (Antal et al., 2005; Traag et al., 2013). The distinction between balanced and imbalanced triads defines an energy function for the network (Marvel et al., 2009), providing a framework for analyzing network stability and dynamics, reconfiguration, and phase transitions, in addition offering key insights into the adaptability and efficiency of network systems.

Structural balance theory assesses the stability and dynamics of brain networks by analyzing triads of interconnected brain regions (Saberi et al., 2024; Saberi et al., 2021a). Research has identified patterns of balance and imbalance formation in the brain, with cortical regions more frequently participating in balanced triads, while subcortical regions contribute predominantly to imbalanced triadic relations (Saberi et al., 2022). Additionally, balanced configurations are more common within brain subnetworks, whereas imbalanced configurations often appear between different subnetworks, potentially reflecting cross-network integration challenges (Saberi et al., 2022).

The contrast between balance and imbalance, defined in terms of network energy, reflects efficient processing and adaptability in cognitive and sensory systems (Saberi et al., 2024). It also provides insights into brain development quality (Li et al., 2025), as well as the dynamics of emotion processing (Gourabi et al., 2024) and cognitive impairments in neurological disorders (Fakhari et al., 2024; Zamani and Jafadideh, 2024). These findings, along with advancements in predictive modeling (Saberi et al., 2024), suggest that structural balance metrics may serve as biomarkers for neurological and psychiatric conditions, offering predictive tools for examining brain function and dysfunction.

Despite promising studies applying structural balance theory to brain networks, a fundamental question remains unanswered: Does structural balance truly exist between brain regions? Specifically, are triads with a positive product of connections inherently stable, while those with a negative product are unstable and prone to reconfiguration? This core assumption, widely accepted in previous brain studies, lacks direct validation, emphasizing the need for further investigation. While this assumption is well-established in social systems, where structural balance has been supported by experimental studies on relationship dynamics (Morrissette, 1958), its relevance to brain networks remains uncertain. Unlike social interactions, where individuals actively resolve imbalances and conflicts over time (Antal et al., 2005; Gallo et al., 2024; Liu et al., 2020), the mechanisms governing stability and reconfiguration in neural systems may follow different principles. This gap underscores the importance of empirically testing whether structural balance principles apply to brain networks.

To address this fundamental question, we analyzed resting-state fMRI data from healthy adults in the Human Connectome Project (Van Essen et al., 2013), examining the temporal stability and strength of triadic configurations using two defined measures: (i) lifetime, the primary index of triadic stability, and (ii) absolute peak energy, a complementary continuous measure reflecting the strength of agreement or disagreement among the three connections (Saberi et al., 2024). We hypothesized that balanced triads would exhibit longer lifetimes and higher peak energy values, reflecting stable and internally consistent interactions. Conversely, we expected imbalanced triads to display shorter lifetimes and lower peak energy, indicating structural tension and a tendency to reconfigure. To ensure the robustness of our findings, we utilized surrogate signals with randomized phases and compared the lifetime of real brain data to null models, validating that the observed results were not artifacts of random signal fluctuations or network connectivity patterns. Finally, we characterized the occurrence and transition dynamics of each triadic configuration, examining how differences in lifetime between balanced and imbalanced triads influence the prevalence of specific triadic states in the brain.

In the present study, we focus on the temporal persistence (lifetime) of these triadic configurations as the empirical marker of their stability, while energy serves as a complementary measure of their internally consistency.

## Results

We extracted dynamic functional connectivity (Hutchison et al., 2013) from regional brain activations to assess the stability and strength of triadic configurations in functional brain networks (Figure 1A). Specifically, we tracked the temporal dynamics of each triangle formed by three distinct brain regions across consecutive time windows (Figure 1C). A given triangle could appear in different triadic states and transition between them over time. For each occurrence of a triadic state, we computed two key measures: (1) lifetime, defined as the continuous duration during which the specific triadic configuration persisted, and (2) absolute peak energy, defined as the maximum absolute value of the triad’s energy during its lifetime.

These measures were averaged across all occurrences in all triangles of brain regions for each triadic state in each subject’s dynamic functional connectivity. As a result, we obtained one average lifetime and one average peak energy value for each of the four possible triadic states per subject, which were then used for group-level analyses.

### Imbalanced triads show shorter lifetimes than balanced triads

To test our hypothesis that imbalanced triadic configurations are less stable than balanced ones, we compared the lifetimes of the four possible triadic states across subjects (Figure 1D). As predicted, imbalanced triads (−−− and +−+) exhibited significantly shorter lifetimes compared to balanced configurations (+++ and −+−). The two imbalanced triads showed reduced temporal persistence, with the shortest lifetime observed in the triad containing three negative connections (−−−). In contrast, balanced triads demonstrated notably longer lifetimes, with the two-negative/one-positive triad (−+−) exhibiting the highest temporal stability.

These findings support the assumption that imbalanced triads, which introduce structural tension in the network, are inherently unstable and more prone to reconfiguration. Conversely, balanced configurations reflect more enduring patterns of structural consistency. The consistent difference in temporal persistence between balanced and imbalanced triadic types highlights the validity of applying structural balance theory to brain networks (see Supplementary Table 1 for group-level comparisons and effect sizes).

### Balanced triads reach higher peak energy than imbalanced triads

We next examined whether momentary internally consistency strength of triads, quantified by their absolute peak energy, differed between balanced and imbalanced configurations (Figure 1E). Consistent with our hypothesis, balanced triads (+++ and −+−) exhibited significantly higher absolute peak energy values than imbalanced ones (−−− and +−+). This pattern indicates that balanced triads are characterized by stronger and more internally consistent interactions among their constituent regions. Among the balanced configurations, the two-negative/one-positive triad (−+−) reached the highest peak energy values, suggesting it represents the most internally consistent configuration. In contrast, imbalanced triads showed significantly lower peak energy, with the lowest values observed in the triad composed of three negative connections (−−−).

These results reinforce the interpretation that imbalanced triads not only persist for shorter durations but also fail to reach higher energy levels due to dissonance in inter-regional interactions, an outcome consistent with the core assumptions of structural balance theory (see Supplementary Table 2 for group-level comparisons and effect sizes).

### Lifetime-peak energy associations reflect (strong) structural balance classes

We examined the association between triad lifetime and absolute peak energy for each triadic state (Figure 1F). The joint distribution revealed two distinct clusters corresponding to balanced and imbalanced triads, highlighting a functional divergence between these two classes and functional similarity within each class. Balanced triads were characterized by longer temporal persistence and stronger coordination among brain regions, whereas imbalanced triads were more transient and energetically weaker. Within each class, triadic subtypes showed similar profiles, supporting the validity of their classification under (strong) structural balance theory.

Notably, imbalanced triads exhibited a linear relationship between peak energy and lifetime $\left(y=0.012x-0.002\right)$ , suggesting a constrained and proportional dynamic. In contrast, balanced triads followed a nonlinear pattern, in which increases in lifetime were associated with relatively lower peak energy increase $\left(y=-0.0002x^{2}+0.015x-0.039\right)$ . These results further underscore the valid classification of four triad types defined by (strong) structural balance theory in brain networks.

### Triadic lifetimes in brain networks deviate from random models

**FIGURE 2:**
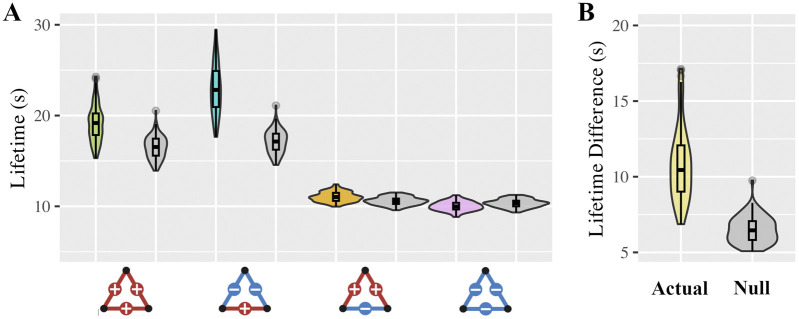
Comparison of triadic lifetime patterns in brain networks versus surrogate models. **(A)** Violin plots show the group-level distribution of triad lifetimes for each triadic configuration in real brain networks (colored) and phase-randomized surrogate networks (gray), highlighting the divergence between real and null values. **(B)** Violin plot comparing the difference in lifetime between balanced and imbalanced triads in actual versus surrogate networks. The greater difference observed in the real data suggests that triadic stability reflects structured network coordination in the brain rather than random fluctuations.

To ensure that the observed lifetime patterns were not driven by the random organization of brain connections, we compared the lifetime of each triadic configuration in the actual brain data to corresponding values from surrogate models (Váša and Mišić, 2022; Prichard and Theiler, 1994) constructed using randomized phase signals (Figure 2A).

Across subjects, we observed significant differences from the null distributions for all triadic types. Specifically, three of the four triad types: the fully positive triad (+++), the mixed triad with two positive and one negative connection (+−+), and the mixed triad with one positive and two negative connections (−+−), exhibited significantly longer lifetimes in the actual data compared to their surrogate models. This suggests that these configurations are more stable than would be expected by chance. In contrast, the fully negative triad (−−−) showed shorter lifetimes in the actual brain data than in the null model, indicating that this imbalanced configuration is particularly unstable and may be actively avoided in the brain’s functional dynamics. Supplementary Table 3 demonstrates group-level comparisons and effect sizes.

To further assess the distinctiveness of triadic configurations in real versus surrogate networks, we compared the lifetime difference between balanced and imbalanced triads across the two models (Figure 2B). The significantly greater lifetime difference observed in the real data relative to the surrogate models (non-parametric p-value <0.001, effect size = 0.96) supports the conclusion that the formation and persistence of structural balance in brain networks reflect meaningful, non-random dynamics rather than stochastic fluctuations.

### The distribution of triadic occurrence is non-uniform

**FIGURE 3:**
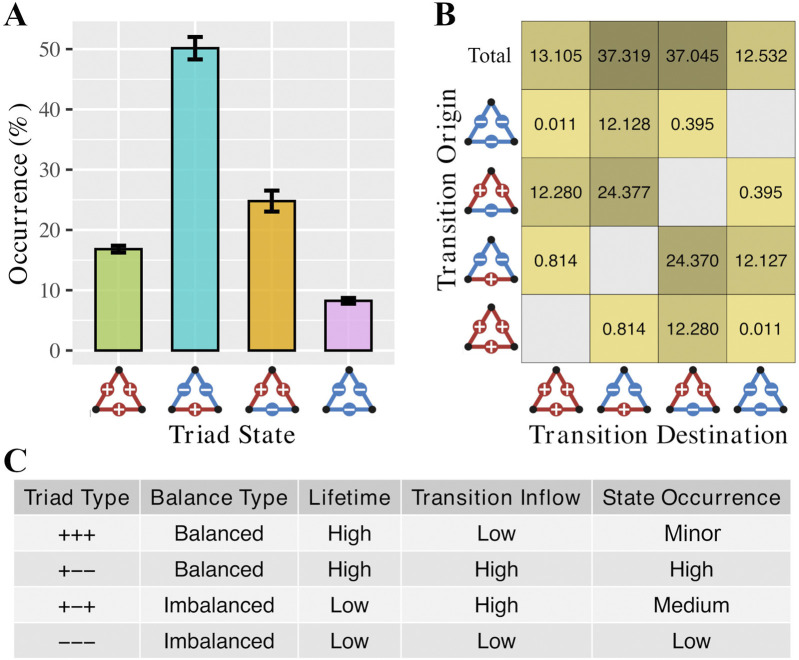
Occurrence and transition dynamics of triadic configurations in brain networks. **(A)** Bar plot showing the non-uniform group-level occurrence of each triadic configuration across all triangles and time windows. **(B)** Transition matrix displaying the average proportion of state changes between triadic configurations, highlighting the prominent role of intermediate states. **(C)** Summary table linking each triad type’s lifetime and transition inflow to its overall state occurrence, suggesting that both temporal persistence and transition dynamics jointly shape the prevalence of triadic states in functional brain networks.

To understand how the lifetime of distinct triadic configurations affects their occurrence in the dynamics of brain networks, we first quantified the frequency of each triad type across all triangles and time windows for each subject, followed by group-level comparison (Figure 3A).

Among the four possible triadic states, the balanced mixed-sign triad (−+−) was the most frequently observed, followed by the imbalanced mixed triad (+−+), the fully positive balanced triad (+++), and finally the fully negative imbalanced triad (−−−), which was the least frequent. This pattern suggests that certain triadic configurations are more frequently expressed in functional brain dynamics, potentially due to their stability and transition characteristics.

### Intermediate configurations are preferred in triadic state transitions

We next investigated how transitions occur between different triadic configurations over time by assessing the transition proportions between each pair of triad types (Figure 3B). Transition patterns were not uniformly distributed: balanced triads often transitioned into imbalanced triads and vice versa. Notably, the balanced −+− triad and the imbalanced +−+ triad exhibited both high outflow and high inflow, indicating active role of intermediate states. In contrast, the fully positive triad (+++) and fully negative triad (−−−) showed low transition, both entering and exiting. Overall, triads were more likely to transition into −+− and +−+ configurations than into the other two states.

### Lifetime and transition inflow determine triadic state prevalence

Together, recent findings combined with earlier results on triadic lifetimes, clarify how transition patterns and temporal stability shape the prevalence of each triad type. The −+− triad, characterized by both high lifetime and high transition inflow, emerged as the most frequently occurring state. The +++ triad, while exhibiting high stability, showed lower transition inflow, leading to a minor level of occurrence. Additionally, the +−+ triad, with lower lifetime but high transition inflow, reached a medium level of occurrence. Finally, the −−− triad, with both low lifetime and low transition inflow, was the least frequent configuration. These dynamics reinforce the conclusion that both temporal persistence and transition architecture are key mechanisms driving the expression of structural balance in functional brain networks (Figure 3C).

## Discussion

### Summary

In this study, we investigated the core assumptions of structural balance theory in the context of brain networks. By analyzing dynamic triadic configurations in resting-state fMRI data, we found that balanced triads exhibited significantly longer lifetimes, indicating greater temporal stability, and higher peak energy values, reflecting stronger internal consistency. Together, these complementary measures validate the assumption of structural balance theory that balanced triads represent stable, internally consistent interactions in the brain network, while imbalanced triads are inherently unstable and prone to reconfiguration. These differences formed two distinct clusters in the lifetime-energy space, consistent with the classification of triadic states defined by (strong) structural balance theory. Moreover, lifetime patterns in real data significantly diverged from those observed in surrogate models, confirming that our results are not artifacts of random fluctuations. Finally, specific transition patterns between triadic states, particularly the prominent role of intermediate triads, combined with the lifetime profiles of each triadic state, shaped the overall prevalence of triadic configurations in brain networks.

### Empirical support for the instability of imbalanced and the stability of balanced configurations

The assumptions of structural balance theory, that imbalanced triads are unstable, short-lived, and less frequently observed than balanced ones, have long been supported in social and technological networks. Early experimental work by Morrisette provided foundational behavioral evidence for structural balance in human social interactions (Morrissette, 1958). The study demonstrated that individuals actively resolve imbalanced triads by modifying relationships, reinforcing Heider’s hypothesis that social networks tend to evolve toward balance. Recent studies such as Liu et al. applied structural balance analysis to time-varying signed networks and reported that balanced triads form faster and persist longer, while imbalanced configurations are more transient (Liu et al., 2020). Our findings extend and empirically validate these principles in the context of functional brain networks, revealing that similar balance-seeking dynamics operate in the neural domain.

### Evidence for strong structural balance theory

Two major theoretical formulations exist for structural balance theory: strong structural balance theory and weak structural balance theory. The strong version, based on the foundational work of Heider and formalized by Cartwright and Harary (Heider, 1946; Heider, 2013; Cartwright and Harary, 1956), posits that the triadic configurations +++ and −+− are structurally balanced, while +−+ and −−− are imbalanced. The weak version, proposed by Davis (Davis, 1967), generalizes this concept by emphasizing dyadic alliance consistency and allowing for a greater variety of stable group structures. It treats −−− triads as balanced and considers −+− the only imbalanced configuration. Recent empirical work by Gallo et al. provides support for both versions (Gallo et al., 2024). Our findings, in the context of functional brain networks, align with the strong version of structural balance theory. Specifically, the joint distribution of triad lifetime and peak energy revealed two distinct clusters, separating the +++ and −+− configurations from the −−− and +−+ triads. Balanced triads exhibited greater temporal persistence and higher interaction energy, whereas imbalanced triads were more transient and exhibited weaker energetic signatures.

### Filling the gap in structural balance analysis of brain networks

Structural balance theory has become an increasingly valuable framework in the study of functional brain networks, with a wide range of applications yielding both theoretical and clinically meaningful insights. For example, balanced configurations are more frequently observed within brain subnetworks, whereas imbalanced triads tend to occur across networks, often involving subcortical regions contributing to triadic frustration across distinct canonical systems (Saberi et al., 2022). Imbalanced triads have been shown to be least frequent in early adulthood compared to both earlier and later life stages, suggesting that increased structural balance is associated with neural maturation and reduced inter-regional conflict (Saberi et al., 2021b).

Other studies have investigated how the topology of positive and negative links contributes to the contrast between balanced and imbalanced triads, quantified as network energy, and have shown that the resting-state brain tends toward a more stable, low-energy configuration (Saberi et al., 2021a). Network energy has been linked to efficient sensory processing and cognitive adaptability, particularly during tasks involving cognitive control (Saberi et al., 2024), and has also been observed to increase during emotional processing (Gourabi et al., 2024). Differences in network energy have also been reported in developmental contexts, such as greater flexibility and higher energy in full-term compared to preterm infants, especially in the visual, sensorimotor, and frontoparietal networks (Li et al., 2025). In clinical populations, higher ADHD symptom severity has been associated with lower network energy, indicating a shift toward more rigid, less flexible brain states (Fakhari et al., 2025). Similarly, altered balance energy has been found in individuals with obsessive-compulsive disorder and autism spectrum disorders (Talesh et al., 2023; Moradimanesh et al., 2021), and structural balance theory has been applied to assess network responses in rTMS stimulation (Kashyap et al., 2023; Ansari Esfeh et al., 2025).

Despite these productive and wide-ranging applications, a fundamental gap remains: the core assumptions of structural balance theory, that balanced triads are more stable while imbalanced triads are unstable and transient, have never been directly tested in the brain. Previous studies have largely relied on statistical interpretations of triad distributions or global energy patterns, without empirically validating stability assumptions of triadic configurations. Our study addresses this critical gap by providing empirical validation of structural balance theory in functional brain networks, using dynamic measures of triad lifetime and peak energy. By validating the assumptions that prior studies have implicitly adopted, our results support the theoretical grounding for applying structural balance theory to understand the brain’s functional architecture.

### A dynamic framework for investigating structural balance in brain networks

To date, all applications of structural balance theory in neuroscience have been conducted using static functional connectivity, typically computed across an entire resting-state or task session (Saberi et al., 2024; Saberi et al., 2022; Li et al., 2025; Gourabi et al., 2024; Fakhari et al., 2025; Zamani and Jafadideh, 2024; Saberi et al., 2021b; Talesh et al., 2023; Moradimanesh et al., 2021; Kashyap et al., 2023; Ansari Esfeh et al., 2025). This approach examines the presence or frequency of balanced and imbalanced triads in a fixed network, providing useful descriptive insights into the global organization of brain connectivity. In contrast, our study adopts a dynamic functional connectivity framework (Hutchison et al., 2013), allowing us to reconstruct triadic states and track the evolution of structural balance over time.

This shift from static to dynamic analysis represents both a conceptual and methodological advance, enabling the direct examination of how triads emerge, persist, and transition throughout ongoing brain activity. We introduced two key metrics, triadic lifetime and peak energy, to capture the temporal stability and interaction strength of triadic configurations. Additionally, we analyzed the transitions between triadic states, revealing dynamic reconfiguration patterns that are not accessible through static methods.

Together, these measures go beyond static triad counts, offering a richer, temporally resolved understanding of balance as a dynamic, state-dependent phenomenon. This framework opens new possibilities for studying not only the stability of brain states but also the pathways and mechanisms of network reorganization in response to internal or external demands. It provides a powerful tool for mapping neural stability, adaptability, and dysfunction in both healthy and clinical populations. Furthermore, this approach can help uncover the role of higher-order interactions in shaping the dynamic architecture of functional brain systems.

### Rationale for dataset selection

The choice of dataset plays a critical role in addressing specific research objectives. For this study, we selected resting-state fMRI data from the Human Connectome Project (HCP) (Van Essen et al., 2013), a highly standardized and widely used resource in neuroimaging research. This dataset offered several key advantages that made it particularly well-suited to our goal of empirically validating the fundamental assumptions of structural balance in the brain.

First, the HCP data were collected from healthy young adults, minimizing the influence of developmental or clinical confounds and allowing us to focus on brain network organization. Second, all scans were acquired using the same scanner model, eliminating inter-scanner variability and ensuring consistency across participants. Third, the use of a robust, ICA-based parcellation scheme derived from group-level analysis provided functionally meaningful brain regions while supporting reproducibility. Fourth, the dataset underwent a uniform and high-quality preprocessing pipeline, reducing artifacts and enhancing the reliability of dynamic connectivity measures. Finally and critically, the extended scan duration characteristic of the HCP allowed us to effectively capture the brain’s temporal fluctuations in connectivity, which are essential for our time-resolved triadic analysis.

Now that the methodological framework and key measures, triadic lifetime, peak energy, and state transitions, have been developed and validated using a clean, normative dataset, this approach can be readily applied to a wide range of datasets, including those involving clinical populations, developmental cohorts, and task-based paradigms.

### Surrogate modeling for testing dynamic structural balance

To explore whether the observed lifetime results of triad configurations in brain networks reflect meaningful functional organization or could arise by chance, we implemented a phase-randomized surrogate modeling approach at the level of regional brain time series (Váša and Mišić, 2022; Prichard and Theiler, 1994). This technique preserves key features of the original signals, such as temporal autocorrelation, power spectrum, and overall amplitude distribution, while disrupting phase relationships that give rise to higher-order network structure. This approach is particularly well-suited to our study, as we investigated the dynamic properties of brain networks.

In contrast, previous applications of structural balance theory in brain networks have typically relied on topology-based null models, such as random edge rewiring or models that preserve certain aspects of the network’s static architecture (e.g., degree sequence, edge sign distribution) (Saberi et al., 2024; Saberi et al., 2022; Saberi et al., 2021a). These models are appropriate for evaluating structural balance in static networks but are insufficient for testing dynamic hypotheses about network evolution and stability. In those studies, such topology-based randomization demonstrated that structural balance in static brain networks is not a random effect. Building on this foundation, the present work extends the framework into the temporal domain, where surrogate modeling provides a principled way to test whether observed lifetimes and energy patterns arise from genuine neural coordination rather than stochastic fluctuations.

## Limitations and future directions

While our study introduces a dynamic framework for validating structural balance theory in brain networks and provides evidence supporting its core assumptions, several limitations should be addressed in future studies.

First, the analysis was conducted using a sample of healthy young adults from the Human Connectome Project. While this choice enabled control over confounding variables such as age and clinical status, it also limits the generalizability of our findings to more diverse populations, including children, older adults, and individuals with psychiatric or neurological conditions. Future research should apply this framework to developmental, aging, and clinical cohorts to explore how triadic dynamics vary across different stages of life and health conditions.

Second, although our phase-randomized surrogate modeling approach provides a rigorous temporal null model, it does not incorporate spatial constraints or network-level properties beyond pairwise dependencies. Future studies could expand surrogate models to include modularity constraints or higher-order topological controls to refine null comparisons further.

Third, while this study focused on resting-state fMRI, applying the same framework to task-based data could help clarify how triadic balance shifts during cognitive engagement, emotional processing, or sensory integration. Similarly, combining this approach with behavioral, clinical, or genetic measures could illuminate the functional relevance of triadic balance dynamics in individual variability and brain-behavior relationships.

## Conclusion

This study provides the first empirical validation of the core assumptions of structural balance theory in brain networks, demonstrating that imbalanced triads are intrinsically unstable and conflictual, while balanced triads exhibit stability and internally consistent interactions. Our findings revealed a clear separation between two classes of triadic configurations, providing support for the strong version of structural balance theory in the context of brain network. The results significantly diverged from those expected under random conditions, confirming that the observed findings were not artifacts. Moreover, distinct transition dynamics and lifetime profiles of each triadic state shaped their overall occurrence, with intermediate triadic types playing a prominent role in network configuration. By introducing a novel framework to study the temporal dynamics of triadic brain interactions, along with tools such as triadic lifetime, peak energy, and transition mapping, this work opens a promising avenue for exploring the role of higher-order network organization in brain function, cognition, and dysfunction.

## Methods

### Dataset

We utilized resting-state fMRI (rfMRI) data from the Human Connectome Project (HCP) S1200 release, a high-quality and publicly available neuroimaging dataset designed to support large-scale, reproducible brain research (Van Essen et al., 2013). Specifically, we analyzed data from 100 healthy participants (54 females, 46 males), selected from a larger cohort of 1,200 subjects. Participants were young adults, primarily between 22 and 35 years of age, with the following age distribution: 17 in the 22–25 range, 40 in 26–30, 42 in 31–35, and 1 above 36 years old. This subset included only individuals with no known familial relationships, thereby reducing genetic confounds and increasing the generalizability of group-level inferences.

Each participant completed four 15-min rfMRI runs, yielding a total of 4,800 volumes per subject. Data were collected with a multiband EPI sequence at a temporal resolution of TR = 720 ms, offering a high temporal sampling rate suitable for dynamic functional connectivity analysis. All data underwent HCP’s minimal preprocessing pipeline, which included spatial normalization, intensity normalization, and motion correction (Glasser et al., 2013). Artifacts were removed using the ICA + FIX pipeline (FMRIB’s ICA-based X-noiseifier), a machine-learning classifier trained to distinguish noise components from neural signal (Salimi-Khorshidi et al., 2014; Griffanti et al., 2014). Cross-subject alignment of cortical surfaces was achieved using MSMAll (Multimodal Surface Matching) based on areal features, improving spatial correspondence across individuals (Robinson et al., 2014; Glasser et al., 2016).

To extract brain region time series, HCP group-PCA and group-ICA pipeline were utilized. First, subject data were temporally demeaned and variance-normalized, and group-PCA was conducted using MIGP (MELODIC’s Incremental Group-PCA), which approximates concatenated PCA on all subjects (Smith et al., 2014). The PCA eigenmaps were then decomposed using FSL’s MELODIC to perform spatial independent component analysis (ICA) in grayordinate space (Smith et al., 2014; Beckmann and Smith, 2004; Hyv and arinen, 1999). The HCP provides multiple group-ICA decompositions at different spatial resolutions (ICA25, ICA50, ICA100, ICA200), corresponding to increasingly fine-grained representations of functional organization. We used the 50-component (ICA50) parcellation, which offers an optimal balance between spatial resolution, reproducibility, and functional interpretability, while remaining computationally tractable for dynamic analyses. This decomposition yielded 50 spatially independent brain components, interpretable as functional nodes.

For each subject, node time series was extracted using dual regression (stage 1) (Filippini et al., 2009). In this step, the 50 ICA spatial maps were regressed onto each individual’s preprocessed rfMRI data, yielding 50 representative time series per subject. Each time series consisted of 4,800 timepoints, capturing temporally resolved neural activity across distributed brain regions. These regional time series served as input for all subsequent analyses of dynamic connectivity and triadic structural balance.

Importantly, the use of group-level ICA50 parcellation and MSMAll alignment ensured one-to-one correspondence of functional nodes across all subjects. Each element of the 50 × 50 connectivity matrix therefore represents the same pair of brain regions for every participant. This standardized parcellation enables valid cross-subject comparison of dynamic metrics such as triadic lifetime and energy, which are derived from the same spatially homologous network structure.

### Dynamic brain network formation

To characterize the temporal evolution triadic configurations between brain regions, we initially constructed dynamic brain networks using a sliding window approach (Hutchison et al., 2013). As noted above, node identities were consistent across subjects due to the use of a common ICA50 parcellation. For each subject, we applied a sliding window of 50 timepoints (equivalent to 36 s given TR = 720 ms) across the full-length time series (4,800 timepoints; corresponding to approximately 3,456 s or 57.6 min across four runs). The slight difference from the nominal 60-min duration reflects the HCP’s internal acquisition structure. No additional volumes were discarded before our analysis, as the HCP minimal preprocessing pipeline already removes initial non–steady-state images.

The window advanced continuously with a step size of one timepoint, generating a series of temporally overlapping windows. Within each window, we extracted a segment of activation time courses from the 50 ICA-derived brain regions. Functional connectivity was estimated by computing the Pearson correlation between all pairs of regional activation time courses in each window. This yielded a 50 × 50 connectivity matrix per window, where each matrix cell represents the strength and sign of the correlation between two regions. Both positive and negative correlations were preserved to capture co-activations and anti-correlations between regional brain activations. Each connectivity matrix was interpreted as the adjacency matrix of a weighted signed graph, where nodes represent brain regions, edges correspond to functional connections. As the sliding window progressed, the sign and strength of connections evolved, resulting in a time-resolved sequence of connectivity matrices for each subject. The output of this procedure was a three-dimensional array per subject, with dimensions 50 × 50 × T, where T corresponds to the number of sliding windows (Figure 1A). This array constitutes the dynamic functional network, capturing the temporal evolution of brain connectivity throughout the scan and serving as the input for triadic and structural balance analyses.

### Structural balance theory

Structural balance theory provides a theoretical framework for analyzing the stability and organization of signed networks by focusing on triadic configurations (Saberi et al., 2024; Estrada, 2019; Marvel et al., 2009). Originally introduced in the context of social relationships, the theory posits that the relationship between any two nodes is influenced by their mutual connection to a third node (Heider, 2013; Cartwright and Harary, 1956). In a signed network where edges carry positive or negative weights, each triangle can be classified into one of four possible triadic configurations based on the combination of edge signs: all connections are positive (+++), two positive edges and one negative (+−+), two negative edges and one positive (−+−), all connections are negative (−−−). According to the (strong) structural balance theory, these four configurations are further grouped into two categories based on their structural tension and theoretical stability: Balanced triads: +++ (“the friend of my friend is my friend”) and −+− (“the enemy of my enemy is my friend”) are considered stable configurations that reflect internally consistent relationships. No node is motivated to change the quality of its connections. Imbalanced triads: +−+ (“my friend is friends with my enemy”) and −−− (“everyone is enemies with each other”) introduce structural tension or dissonance, reflecting inconsistency among the relationships. According to balance theory, imbalanced triads are inherently unstable and tend to evolve toward balanced states over time.

To translate these theoretical notions into measurable properties of brain network dynamics, we modeled stability in the temporal domain through the triadic lifetime, the duration for which a specific triadic configuration persists continuously across sliding-window time frames. Within this framework, a longer lifetime indicates that the configuration resists reorganization and thus reflects greater stability, whereas a shorter lifetime signifies a transient or unstable state. Accordingly, balanced triads are expected to display longer lifetimes, consistent with their theoretical resilience to perturbation, while imbalanced triads should exhibit brief, rapidly changing lifetimes, capturing their instability. This temporal measure provides an empirical bridge between the conceptual notion of structural balance and the dynamic evolution of functional interactions in the brain.

Triad energy quantifies the internal tension or consistency of a triad formed between i , j , and k in a continuous manner as follows (Saberi et al., 2024):

$$E_{\Delta }=-\sqrt[{3}]{S_{ij}\times S_{ik}\times S_{jk}}$$

where $S_{ij}$ represent the weight between nodes i and j , respectively. The cubic root ensures the resulting energy value is dimensionless and less sensitive to the magnitude of individual links. The negative sign makes the metric “physics-friendly,” assigning lower (more negative) energy values to balanced configurations and higher (more positive) energy values to imbalanced configurations. Because correlation values range between −1 and 1, the energy of a triangle also ranges continuously between −1 and 1. This continuous measure complements the categorical classification of triads.

Importantly, this continuous energy measure complements rather than replaces the categorical balance classification and the temporal lifetime metric. While lifetime directly quantifies the stability of triadic states across time, energy characterizes their momentary internally consistency strength. Together, these measures provide a joint description of triadic dynamics, how strongly and how persistently each configuration manifests in the brain’s functional network.

### Triadic configuration analysis

To investigate structural balance dynamics in functional brain networks, we performed a triadic analysis across time-resolved functional connectivity matrices derived from dynamic brain networks. Given the 50 ICA-derived brain regions used in our analysis, we considered all possible combinations of three distinct nodes, resulting in $\left(\begin{array}{l} 50\\ 3 \end{array}\right)=19600$ unique triangles per subject.

Each triangle was characterized by three continuously varying edge weights representing the pairwise functional connections between the three regions. The signs of these connections determined the triadic configuration at each time point (i.e., +++, +−+, −+−, or −−−), while the product of the three edge weights defined the triadic energy, reflecting the strength and consistency of the configuration.

As brain activity evolved over time, the configuration and energy of each triangle changed accordingly. To quantify these dynamics, we introduced two key measures for each period in which a triangle maintained a specific triadic state: lifetime as the number of consecutive time windows during which a triangle remained in the same triadic configuration; absolute peak energy: the maximum absolute value of the triadic energy during that lifetime period.

Each triangle could undergo multiple transitions between triadic states over time and reach different peak energy values within each state episode. We aggregated all lifetimes and peak energy values for each of the four triadic types (+++, +−+, −+−, −−−) across all 19,600 triangles and all time windows for each subject. This yielded one average lifetime and one average peak energy per triadic type per subject, resulting in a total of four lifetime and four peak energy values per subject.

For transition analysis, we tracked every change in triadic state over time for each triangle and recorded the number of transitions from one triadic type to another. These transitions were aggregated across all triangles and over time, producing a transition count matrix representing the directional frequency of state changes. For triadic occurrence, we counted the total number of times each triadic configuration appeared across all triangles and time windows per subject.

These measures were calculated at the subject level and used for subsequent group-level statistical analyses.

### Surrogate modeling for null network generation

To determine whether the observed patterns of triadic stability in brain networks were meaningful or could arise by chance, we employed a surrogate modeling approach that preserved key statistical properties of the data while disrupting their temporal dependencies (Váša and Mišić, 2022). This method allowed us to construct null dynamic networks for comparison with real brain networks.

Unlike topology-based random networks, which are suitable for static graph analyses but do not capture time-dependent reconfiguration, we adopted a phase-randomization approach that operates directly at the signal level. This method is particularly appropriate for dynamic analyses, as it preserves each region’s amplitude distribution, power spectrum, and autocorrelation while eliminating the phase relationships responsible for functional coupling. The surrogate modeling provides a more realistic control for temporal organization in continuously evolving brain networks.

For each subject, we manipulated the original regional time series using a phase-randomization procedure (Prichard and Theiler, 1994). Specifically, each time series was first transformed from the time domain into the frequency domain using the Fast Discrete Fourier Transform (FFT). In the frequency domain, we retained the original amplitude spectrum to preserve the power distribution across frequencies and randomized the phase of each frequency component. We then reconstructed a surrogate complex signal by combining the original amplitudes with the randomized phases. An inverse FFT was applied to convert the surrogate complex signals back into the time domain, yielding surrogate time series that retained the same autocorrelation structure and power spectrum as the original signals but lacked meaningful phase relationships.

Using these surrogate time series, we constructed surrogate dynamic functional connectivity networks following the same sliding window and correlation procedures applied to the original data. For each subject, ten independent surrogate realizations were generated, and the resulting lifetime and peak energy values were averaged to obtain a stable null reference.

### Statistical analysis

To examine group-level differences in lifetime and absolute peak energy between the four triadic configurations, we used nonparametric Wilcoxon signed-rank tests for all possible pairwise comparisons. Effect sizes were computed using Cliff’s delta, and False Discovery Rate (FDR) correction was applied to control for multiple comparisons.

To examine whether triadic lifetime patterns in real data differed from those expected under chance, we compared the lifetime of each triad type in real networks with its corresponding value in phase-randomized surrogate networks using Wilcoxon signed-rank tests, corrected using FDR. We also performed this test to compare the difference in lifetime between balanced and imbalanced triads in the real data versus surrogate data.

All statistical analyses were conducted using R software along with several supporting packages for data processing, statistical testing, and figure generation (R Core Team, 2020; Torchiano, 2020; Wickham, 2016). Schematic illustrations were created using BioRender.com.

## Data Availability

The original dataset is publicly available at https://db.humanconnectome.org/. All triadic analysis code are openly accessible on GitHub at https://github.com/majidsaberi/StructuralBalance Dynamics, enabling full reproducibility and encouraging further development and application of our framework.
