# Supplementary material for: Empirical evidence for structural balance theory in functional brain networks
Source: Front Netw Physiol. 2026 Jan 16;5:1681597. doi: 10.3389/fnetp.2025.1681597 (PMC12855509; doi:10.3389/fnetp.2025.1681597)
Supplement: Supplementary file 1 [file Supplementaryfile1.docx]

**Supplementary Materials**

**Empirical Evidence for Structural Balance Theory in Functional Brain Networks**

**Authors**

Majid Saberi^1, 2*^, Abolfazl Haqiqifar^3^, AmirHussein Abdolalizadeh^4^, Bratislav Misic^5, 6^, Ali Khatibi^7, 8*^

**Affiliations**

1. Department of Biologic and Materials Sciences & Prosthodontics, University of Michigan School of Dentistry, Ann Arbor, MI, USA
2. Neurosciences & Mental Health Program, The Hospital for Sick Children Research Institute, Toronto, Canada
3. Department of Physics, Faculty of Science, Bu-Ali Sina University, Hamedan, Iran
4. Biological Psychology, Department of Psychology, School of Medicine and Health Sciences, Carl Von Ossietzky Universität Oldenburg, Oldenburg, Germany
5. Department of Neurology and Neurosurgery, McGill University, Montreal, QC, Canada
6. McConnell Brain Imaging Centre, Montreal Neurological Institute and Hospital, Montreal, QC, Canada
7. Centre of Precision Rehabilitation for Spinal Pain (CPR Spine), School of Sport, Exercise and Rehabilitation Sciences, University of Birmingham, Birmingham, UK
8. Department of Psychology, University of Bath, Bath, UK.

* **Corresponding Authors:**

[majidsa@umich.edu](mailto:majidsa@umich.edu)

[ali.khatibi@gmail.com](mailto:ali.khatibi@gmail.com)

|  |  | Balance | | Imbalance | |
| --- | --- | --- | --- | --- | --- |
|  |  | (+ + +) | (+ - -) | (+ + -) | (- - -) |
| Balance | (+ + +) | - | 0  (0.75) | 0  (1) | 0  (1) |
|  | (+ - -) | - | - | 0  (1) | 0  (1) |
| Imbalance | (+ + -) | - | - | - | 0  (0.82) |
|  | (+ + +) | - | - | - | - |

**Supplementary Table 1.** Lifetime comparison between four triad types. Cells indicate corrected non-parametric p-values, and the Cliff's Delta effect sizes are denoted inside parentheses.

|  |  | Balance | | Imbalance | |
| --- | --- | --- | --- | --- | --- |
|  |  | (+ + +) | (+ - -) | (+ + -) | (- - -) |
| Balance | (+ + +) | - | 0  (0.51) | 0  (1) | 0  (1) |
|  | (+ - -) | - | - | 0  (1) | 0  (1) |
| Imbalance | (+ + -) | - | - | - | 0  (0.53) |
|  | (+ + +) | - | - | - | - |

**Supplementary Table 2.** Peak Absolute Energy comparison between four triad types. Cells indicate corrected non-parametric p-values, and the Cliff's Delta effect sizes are denoted inside parentheses.

| Balance | | Imbalance | |
| --- | --- | --- | --- |
| (+ + -) | (+ + +) | (+ - -) | (- - -) |
| 0  (0.73) | 0  (0.97) | 0  (0.45) | 0  (-0.34) |

**Supplementary Table 3.** Lifetime comparison between original network model and surrogate models for four triad types. Cells indicate corrected non-parametric paired p-values, and the Cliff's Delta effect sizes are denoted inside parentheses.
